# The Effect of Lavandula stoechas on Wound Healing in an Experimental Diabetes Model

**DOI:** 10.7759/cureus.45001

**Published:** 2023-09-10

**Authors:** Derya Demir, Ismail Toygar, Emrah Soylu, Ali Tarık Aksu, Aynur Türeyen, Ilgın Yıldırım, Şevki Çetinkalp

**Affiliations:** 1 Pathology, Ege University, Faculty of Medicine, İzmir, TUR; 2 Nursing, Muğla Sıtkı Koçman University, Fethiye Faculty of Health Sciences, Muğla, TUR; 3 Miscellaneous, Ege University, Center for Research on Laboratory Animals, İzmir, TUR; 4 Orthopaedics, Kuki Veterinary Clinic, İstanbul, TUR; 5 Miscellaneous, Ege University, Faculty of Nursing, İzmir, TUR; 6 Diabetes and Endocrinology, Ege University, Faculty of Medicine, İzmir, TUR

**Keywords:** ­wound healing, rat, lavandula stoechas, diabetic foot, diabetes

## Abstract

Introduction: Diabetic foot is a consequential and dangerous complication of diabetes, contributing to decreased quality of life, escalated hospitalizations, and increased mortality rates. Using an experimental model of diabetes, this study aims to investigate the effect of Lavandula stoechas on wound healing.

Methods: A total of 35 albino Wistar rats, 250-350 grams in weight, were used. The rats were divided into five groups, seven rats in each group. Of these, 21 rats were induced with 50 mg/kg streptozotocin (STZ) to mimic the diabetic condition. Additionally, 14 rats remained non-diabetic and served as the control group. The diabetic rats were further divided into three subgroups. The non-diabetic group was split into two subgroups based on the dressing materials used (allicin, physiological serum, and control). Wound dimensions were assessed on Days 0, 7, 14, and 21. Biopsies were taken from the wound sites at the same time.

Results: There were significant differences between groups on Days 7, 14, and 21. The percentage of healing was highest in the Lavandula Stoechas group on Days 7, 14, and 21. Microscopic examination of the biopsies supported accelerated wound healing on Days 7 and 14. Reduced mononuclear cell density and increased hair follicle and adipose tissue development were observed in the DM (diabetes mellitus)-Lavandula Stoechas group on Day 7. On Day 14, the DM-Lavandula Stoechas group increased collagen levels and hair follicles. Similarly, the non-DM-Lavandula Stoechas group showed reduced bullae, dermal edema, and intraepithelial edema on Day 7. This was followed by increased fibroblast levels on Day 14.

Conclusions: In conclusion, this study provides compelling evidence for the potential of Lavandula stoechas extract in the enhancement of diabetic wound healing. The multiple interactions revealed here highlight the need for further investigation into the underlying mechanisms. A cost-effective use of Lavandula stoechas opens up promising prospects in managing diabetic foot healing. This warrants additional research and clinical translation.

## Introduction

Diabetic foot is a common and life-threatening complication of diabetes. The lifelong risk of developing diabetic foot is 15% in diabetes patients. Diabetic foot negatively affects patients by decreasing the quality of life and increasing hospitalization and mortality. Sothornwit et al. (2018) reported that diabetic foot decreases the scores of quality of life among the complications of diabetes [1]. Diabetic foot also increases the healthcare cost and healthcare burden. Diabetes-related lower extremity complications are the leading cause of hospitalization in diabetes patients [2]. The risk of mortality is seven times higher in diabetic foot patients compared to those who do not develop diabetic foot [3]. The five-year mortality of diabetic foot, minor amputation, and major amputation are 30.5%, 46.2%, and 56.6% respectively [4]. During the pandemic, the incidence of diabetic foot, prehospital delay, and mortality increased [5]. Diabetic foot is also the leading cause of nontraumatic lower extremity amputation in most countries [6].

In diabetes patients, the main goal is preventing diabetic foot. However, in the case of the development of the diabetic foot, the goal is decreasing amputation and mortality. The St. Vincent Declaration set a principal target of a 50% reduction of lower limb amputations in diabetes, yet this target has not been achieved [7]. Even though several products are used in the management of diabetic foot ulcers, new products are needed to achieve this target. These new products must be cost-effective and easy to access.

Lavandula stoechas is a common aromatic plant of the Mediterranean region. Lavandula stoechas was reported as an effective approach to improving sleep quality and reducing anxiety in various groups. Lavandula stoechas antibacterial, antifungal, insecticidal, anti-leishmanial, antioxidant, and anti-inflammatory properties were reported in several studies [8]. However, we did not find any studies in the literature investigating the effect of Lavandula stoechas on wound healing in diabetes.

Lavandula stoechas is an easy-to-access, cheap plant that also has antibacterial, antifungal, insecticidal, anti-leishmanial, antioxidant, and anti-inflammatory properties [8]. For this reason, we believe that it may be used in the management of diabetic foot. This study was conducted to investigate the effect of Lavandula stoechas on wound healing in an experimental diabetes model.

## Materials and methods

This randomized controlled study was carried out under in vivo conditions. The experimental protocol was reported according to the ARRIVE (Animal Research: Reporting of In Vivo Experiments) guidelines 2.0 in this section [9].

Hypothesis

H_1_: Lavandula-stoechas extract is an effective agent in accelerating wound healing in diabetic rats.

Study setting

The study was carried out in the Ege University Center for Research on Laboratory Animals between 6 September and 4 October 2021. There wasn’t any blinding in this study.

Study Subjects

In total, 35 (17 males and 18 females) Wistar Albino rats that were 8-12 weeks old and weighing 250-350 grams were used in the study. Cohen’s criteria were used in the determination of the sample size. Accordingly, within the 95% confidence interval limits (α err prob = 0.05), at 0.80 power (1-β) and in the estimated medium effect size (dz=0.65), the minimum required the number of the samples was determined as 35 (seven in each group).

Randomization

For the roughly equal distribution of the males and females in the groups (three males/four females or four males/three females), block randomization was used [10]. Two-step randomizations were used in the study. First, the rats were divided into two groups; diabetic (21 rats) and non-diabetic (14 rats). Then, rats in the diabetic group were induced. After the development of the diabetes model in 21 rats, diabetic rats were divided into three groups; DM (diabetes mellitus)-Lavandula Stoechas, DM-Saline, and DM-Control, and non-diabetic rats were into two groups; non-DM-Lavandula Stoechas and non-DM-Control. A random list generator released by Sealed Envelope Ltd. was used for creating the random lists (Sealed Envelope, London, UK, www.sealedenvelope.com).

Development of diabetes model

The rats in the diabetic groups were induced by intraperitoneal injection of streptozotocin (STZ) dissolved in 0.1M cold sodium citrate buffer, pH 4.5, at a dose of 50 mg/kg. A total of 500 mg STZ dissolved in 100 ml 0.1M cold sodium citrate buffer. The rats’ plasma glucose levels were measured at 24, 48, and 72 hours after induction, and rats with a plasma glucose level of 300 mg/dL were considered diabetic [11, 12].

Wound creation

After inducing the diabetes model, the rats were anesthetized with 75 mg/kg ketamine and 10 mg/kg xylazine injection. The dorsal of the rats were shaved and disinfected with povidone-iodine. Three excisional wounds were then created by full tissue ablation within the panniculus carnosus muscle using a biopsy punch with a 10 mm diameter. Each wound on the dorsal of the rat was at least 1 cm away from the others. After the creation of the wounds, each rat was kept in separate cages to prevent cannibalism [12].

Extraction of Lavandula stoechas

The Lavandula stoechas were bought from the Sepe Natural company. The Lavandula stoechas plants were collected by the company from the southwest of Turkey and extraction was performed with the hydro distillation method. A total of 4 oz. Lavandula stoechas was used in our study. The dominant components of the Lavandula stoechas are camphor and fenkan.

Interventions

The daily dressings were used for rats according to their groups throughout 21 days. Blood glucose measurement, photography of the wound surface, and biopsies collection were administered for the rats in all groups on the baseline, 7th, 14th, and 21st days.

DM-Lavandula Stoechas: The rats in this group were induced with STZ and were diabetic. For the rats in this group, dressing with 0.05 ml Lavandula Stoechas oil was used for 21 subsequent days.

DM-Saline: The rats in this group were induced with STZ and were diabetic. Dressing with 0.05 ml 0.9% NaCl solution was used for 21 subsequent days.

DM-Control: The rats in this group were induced with STZ and were diabetic. There wasn’t any dressing for the rats in this group.

Non-DM-Lavandula Stoechas: The rats in this group were not induced. For the rats in this group, dressing with 0.05 ml Lavandula stoechas oil was used for 21 subsequent days.

Non-DM-Control: The rats in this group were not induced. There wasn’t any dressing for the rats in this group.

Data collection

There were three types of data; descriptive, macroscopic, and microscopic. The descriptive data were plasma glucose levels and weights. Macroscopic data were the wound surface area measurement. On Days 0, 7, 14, and 21, a researcher took a photograph of the wounds with a ruler. These photographs were used in the wound surface measurement by using ImageJ software. Walker formula was used to determine the wound healing percentages [13]. Microscopic data were the biopsies collected from the rats on Days 0, 7, 14, and 21. These tissues were stained with hematoxylin and eosin (H-E) and examined using light microscopy. In the microscopic evaluation, neutrophils, mononuclear cells, intraepithelial edema, dermal edema, fibroblast proliferation, collagen, angiogenesis, epithelialization, skin appendages (hair follicles), and adipose tissue were examined by a blinded pathologist. Epithelialization was evaluated using scale-up: - (null), + (partial), and ++ (complete). Neutrophils, mononuclear cells, fibroblast proliferation, collagen, angiogenesis, epithelialization, skin appendages, and adipose tissue were evaluated using scale-up: - (null), + (weak), ++ (moderate), and +++ (high). Intraepithelial edema and dermal edema were evaluated using scale-up: - (not exist), and + (exist).

Data analysis

Descriptive statistics for the study are presented as numbers (n) and percentages (%). Data were analyzed using parametric tests if the normal distribution assumptions were met and using nonparametric tests if the normal distribution assumptions failed. The results of the parametric tests are given as mean values and standard deviations; the results of the nonparametric tests are given as the median, minimum, and maximum values. Kruskal-Wallis analysis was used in the independent multiple-group comparisons if the normal distribution assumptions failed; the Dunn test was conducted in the post hoc paired comparisons. For dependent multiple-group comparisons, the Friedman test was conducted and the Dunn test was used to determine the pairs that met the significance criteria. p < 0.05 was determined to be the level of significance.

Ethics

Ethical approval from the Ege University Animal Experiment Local Ethical Committee was obtained to carry out the study (approval no: 2018-041). Also, approval from the Ege University Center for Research on Laboratory Animals was obtained.

## Results

Seventeen (48.6%) of the rats were male and 18 (51.4%) were female. There were four females (57.1%) and three males (42.9%) in DM-Lavandula Stoechas, DM-Control, and non-DM-Control groups while four males (57.1%) and three females 42.9%) in DM-Saline and non-DM Lavandula Stoechas groups (Table 1).

**Table 1:** Gender distribution of the rats in the groups

	DM-Lavandula Stoechas	DM-Saline	DM-Control	Non-DM-Lavandula Stoechas	Non-DM-Control	Total
Male	3 (42.9%)	4 (57.1%)	3 (42.9%)	4 (57.1%)	3 (42.9%)	17 (48.6%)
Female	4 (57.1%)	3 (42.9%)	4 (57.1%)	3 (42.9%)	4 (57.1%)	18 (51.4%)
Total	7 (100%)	7 (100%)	7 (100%)	7 (100%)	7 (100%)	35 (100%)

There was a weight loss in diabetic groups while weight gain was in non-diabetic groups. The rats in diabetic groups lost 4.69% of their weight at baseline. On the other hand, there was a 2.55% weight gain in the non-diabetic group compared to the baseline. There were significant differences between the groups on Day 14 (p=0.041) and Day 21 (p=0.008) (Table 2).

**Table 2:** Weight change in groups within time

	DM-Lavandula Stoechas	DM-Saline	DM-Control	Non-DM-Lavandula Stoechas	Non-DM-Control	Test statistics (KW)	p
	X±SS	X±SS	X±SS	X±SS	X±SS		
Baseline	321.90±25.60	317.12±26.14	325.63±22.17	319.15±22.17	322.17±18.64	0.294	0.841
Day 7	315.40±27.73	310.43±21.91	318.61±24.38	321.51±23.49	326.83±21.33	0.359	0.738
Day 14	311.50±25.54	307.32±17.03	312.69±21.34	324.74±26.61	329.58±22.61	1.103	0.041
Day 21	306.60±23.83	303.51±14.96	309.18±19.61	329.55±24.66	328.94±20.39	1.434	0.008

**Figure 1:**
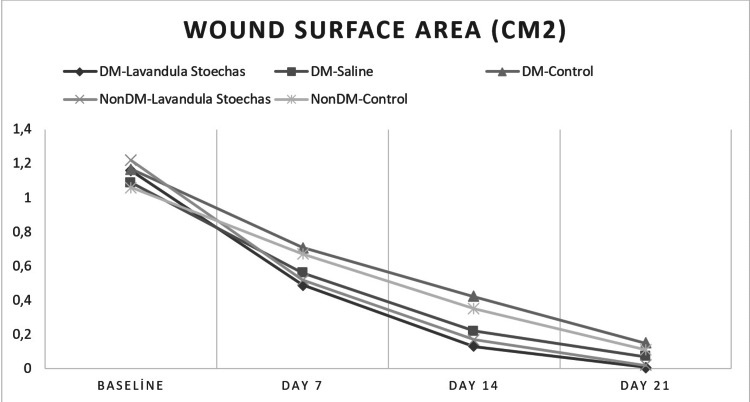
Wound surface area changes over time

There wasn’t a significant difference between the groups at the baseline (p=0.744). However, there were significant differences on Day 7 (p=0.035), Day 14 (p=0.002), and Day 21 (p=0.048) (Table 3). Wound surface change over time is presented in Figure 1.

**Table 3:** Wound surface area (cm2) changes in groups over time

	DM-Lavandula Stoechas	DM-Saline	DM-Control	NonDM-Lavandula Stoechas	NonDM-Control	Test statistics (KW)	p
	X±SS	X±SS	X±SS	X±SS	X±SS		
Baseline	1.16±0.13	1.09±0.11	1.17±0.22	1.22±0.16	1.06±0.15	0.823	0.744
Day 7	0.49±0.09	0.56±0.08	0.71±0.10	0.52±0.09	0.67±0.11	6.129	0.035
Day 14	0.13±0.02	0.22±0.05	0.42±0.09	0.17±0.05	0.35±0.07	14.621	0.002
Day 21	0.01±0.00	0.07±0.01	0.15±0.03	0.02±0.01	0.11±0.06	24.861	0.048

Wound healing percentages were calculated according to the Walker formula. The rank for the wound healing percentages on Day 7 was DM-Lavandula Stoechas (57.76%), Non-DM-Lavandula Stoechas (57.37%), DM-Saline (48.62%), DM-Control (39.32%), and NonDM-Control (36.79%) respectively while the rank for the wound healing percentages on Day 14 was DM-Lavandula Stoechas (88.79%), Non-DM-Lavandula Stoechas (86.07%), DM-Saline (79.82%), Non-DM-Control (66.98%), and DM-Control (64.10%) respectively. On Day 21, the highest wound healing percentage was in DM-Lavandula Stoechas with 99.14% and it is followed by Non-DM-Lavandula Stoechas (98.36%), DM-Saline (93.56%), Non-DM-Control (89.62%), and DM-Control (87.18%) respectively (Table 4).

**Table 4:** Wound healing percentages according to the walker formula

	DM-Lavandula Stoechas	DM-Saline	DM-Control	NonDM-Lavandula Stoechas	NonDM-Control
	%	%	%	%	%
Day 7	57.76%	48.62%	39.32%	57.37%	36.79%
Day 14	88.79%	79.82%	64.10%	86.07%	66.98%
Day 21	99.14%	93.56%	87.18%	98.36%	89.62%

**Figure 2:**
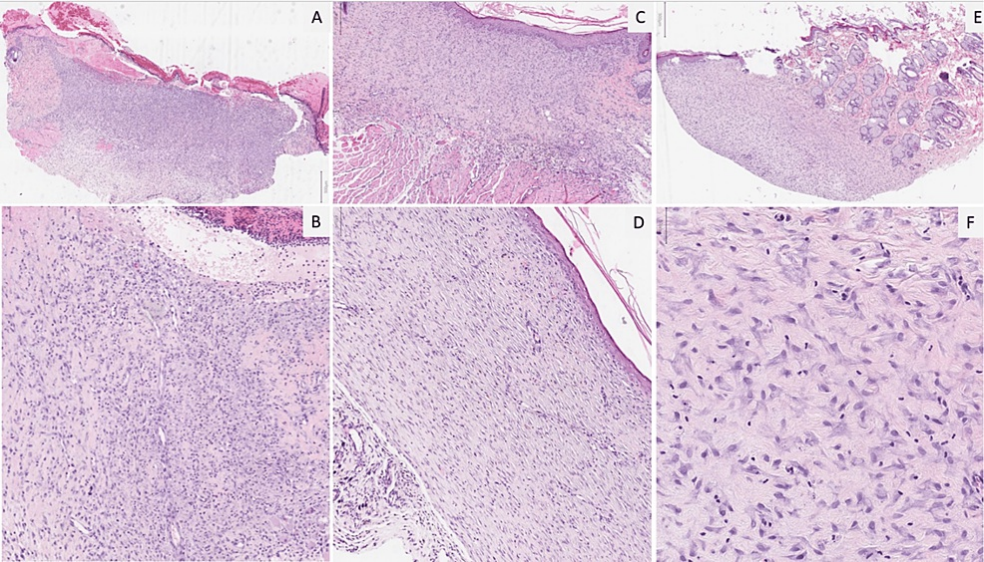
Microscopic images of the wound areas Epithelial loss and ulceration observed in the biopsy on the 7th day (A) (H&Ex20); neovascularization accompanying the ulcer (B) (H&Ex100); on the 14th day, complete regeneration of the epithelium and subepithelial fibroblastic proliferation were showed in DM-Lavandula Stoechas group (C) (H&Ex40); on the 21st day, prominent fibrosis became evident in groups (D) (H&Ex100); by the 14th day, Lavandula Stoechas group showcased increased collagen levels (left side) and hair follicles (right side) (E) (H&Ex20); and higher magnification of collagenization on the left side (F) (H&Ex200).

In the microscopic evaluation of the biopsies collected from the wound area, mononuclear cell density decreased and hair follicle and adipose tissue development were higher in the DM-Lavandula Stoechas group on the 7th day; On the 14th day, when compared to the other groups, the collagen level, and hair follicles were higher in DM-Lavandula Stoechas group; On the 21st day, it was observed that erythema, mononuclear cells, dermal edema, and intraepithelial edema was lower and the number of hair follicles was higher DM-Lavandula Stoechas group. Similarly, in the onn-DM-Lavandula Stoechas group, the level of bullae, dermal edema, and intraepithelial edema was low and the fibroblast level was high on the 7th day; On the 14th day, collagenization, epithelialization, and fibroblast levels were high; on the 21st day, erythema and mononuclear cell density were found to be lower (Figure 2).

## Discussion

In the intricate landscape of diabetes, the convergence of peripheral vascular diseases, hyperglycemia, and neuropathy manifests as a vulnerability that predisposes individuals to diabetic foot and exacerbates the protracted course of wound healing [14]. Diabetic foot, if it is not effectively treated, leads to amputations [15]. Yet, the quest for solutions in this realm is further punctuated by the imperative for affordability. A scarcity of cost-effective materials for dressing diabetic foot ulcers persists, necessitating innovative avenues for intervention. Against this backdrop emerges Lavandula stoechas, an unassuming botanical treasure trove. This easily attainable and economical plant stands as a testament to nature's multifaceted pharmacy, boasting an impressive repertoire of antibacterial, antifungal, insecticidal, anti-leishmanial, antioxidant, and anti-inflammatory properties [8]. A plant of myriad virtues, Lavandula stoechas is a plausible candidate to address the void in diabetic foot ulcer management. Within the contours of this study, the spotlight is cast on Lavandula stoechas' influence on wound healing, woven into the fabric of an experimental diabetes model.

In the purview of this study, meticulous efforts were directed toward ensuring a level playing field for all study groups. The gender distribution, weight, and baseline wound surface were diligently assessed and found to be harmoniously aligned across the groups, substantiating the principle of homogeneity. Statistical analysis underscored the absence of significant discrepancies between the groups, further affirming the congruity.

This study unravels an intriguing facet of the weight dynamics observed within the diabetic groups. It was unmistakably evident that diabetic subjects experienced a discernible weight loss. This phenomenon finds its roots in the intricate orchestration of osmotic diuresis and heightened catabolism, a complex interplay driven by the prevailing hyperglycemia. This metabolic turbulence, a hallmark of diabetes, collectively precipitates weight loss among individuals grappling with the condition [16]. Interestingly, a divergent narrative unfolded within the non-diabetic groups that were the other focus of our study, and these groups exhibited a discernible weight gain. Wistar albino rats exhibit a consistent weight gain pattern until approximately 100 days of age [17, 18]. This attests to these animals' intrinsic dynamics of growth and development. This natural phenomenon imbues our findings with a biological authenticity, grounding our observations in the innate rhythms of the animal model under scrutiny. In the context of our study, it's essential to note that the rats under investigation were in the phase of 8-12 weeks of their Wistar Albino lifespan. During this developmental time, an intrinsic propensity for weight gain is anticipated. This aligns with the inherent growth trajectory exhibited by Wistar Albino rats, a pattern that resonates with their biological propensity for weight accrual during this specific age range. The anticipated weight gain observed in our non-diabetic groups aligns seamlessly with the developmental profile of these rats, thereby reinforcing the reliability and biological relevance of our findings. This convergence with the natural growth trajectory of the rat model lends robustness to our study's observations, firmly anchoring them within the framework of biological norms.

A compelling revelation emerged on the 7th day of our study, where the DM-Lavandula Stoechas group seized the spotlight with the highest percentage of macroscopic wound healing. Remarkably, this commendable outcome was followed by the non-DM-Lavandula Stoechas group. A pivotal insight emerged, delving into the intricate nuances of diabetes-related wound healing; diabetes, renowned for its propensity to prolong inflammatory phases [19], encountered a formidable counterbalance in the form of Lavandula stoechas' anti-inflammatory properties [8]. This alliance seemingly organized a swift truncation of the inflammatory stages, resulting in notably shorter phases compared to their counterparts. Substantiating this situation, the dwindling mononuclear cell density within these groups on the 7th day accentuates the potency of Lavandula stoechas' anti-inflammatory effect, endorsing its role in curtailing the chronicization of inflammatory phases. Moreover, the conspicuous presence of heightened adipose tissue and proliferating hair follicles within the Lavandula Stoechas groups on the 7th day vividly portrays accelerated wound healing. In tandem, the non-DM-Lavandula Stoechas group's elevated fibroblast levels further underscore this narrative of accelerated healing [19]. This symbiotic interplay between Lavandula stoechas' anti-inflammatory attributes and the diabetic wound's proclivity for chronic inflammation unveiled an impressive synergy [20]. The stage was set for accelerated wound healing within these groups, carving a promising trajectory toward restoring tissue integrity and vitality.

The semblance between the outcomes observed on the 7th and 14th days was a striking repetition of the healing process demonstrated by the DM-Lavandula Stoechas group. Once more, this group achieved the highest percentage of macroscopic wound healing, followed by the non-DM-Lavandula Stoechas group on the 14th day. This congruence reaffirms the consistent impact of Lavandula stoechas in driving wound healing, even amid the complex backdrop of diabetes-related challenges. This recurring pattern underscores the potency of Lavandula stoechas' properties and its potential to influence the healing trajectory, regardless of the temporal context. Lavandula Stoechas emerges as a reliable ally in this healing narrative, bridging the gap between diabetic and non-diabetic contexts. Its impact remains steadfast and uniform, unveiling its potential as a potential therapeutic agent in fostering expedited wound healing. Within the 14th day, a rich tapestry of wound healing dynamics unfolded. Notably, the DM-Lavandula Stoechas group exhibited an elevation in collagen levels and a pronounced abundance of hair follicles, creating a vivid tableau of tissue rejuvenation. Simultaneously, the non-DM-Lavandula Stoechas group showcased heightened levels of collagenization, epithelialization, and a flourishing proliferation of fibroblasts, offering a glimpse into the orchestration of the healing process. The complexity of biological mechanisms is a reflection of the multifaceted nature of wound healing. Collagen, a pivotal structural component of tissue, plays a central role in orchestrating tissue repair. Its elevation within the DM-Lavandula Stoechas group is indicative of a robust healing environment where the architectural scaffold is being reinvigorated. Similarly, this group's profusion of hair follicles echoes a revitalization of cellular processes, indicative of a nurturing environment that fosters regeneration. In parallel, the non-DM-Lavandula Stoechas group shines a spotlight on the orchestrated symphony of collagenization, epithelialization, and fibroblast activity. These elements harmonize to fortify the healing trajectory, culminating in a heightened tissue repair and restoration capacity. This ensemble of healing factors is orchestrated with precision, underpinned by Lavandula stoechas' potential to catalyze healing cascades, even in the complex context of diabetes. The delicate balance of wound healing unfolds against intricate cellular structures. The inflammatory cytokines play a pivotal role in managing the initial inflammatory phases of the healing process. However, their prolonged presence can tip the scales, steering the course toward complex challenges. This includes impaired angiogenesis, a vital process for nurturing new blood vessels, as well as inhibited production of fibronectin and type I collagen, essential building blocks of tissue regeneration. Notably, this prolonged inflammatory milieu can also trigger the release of proteolytic enzymes, including matrix metalloproteases, which, while necessary in moderation, can become detrimental when their secretion goes unchecked [19, 21].

In parallel, the stage set by diabetes introduces another challenge overproduction of reactive oxygen species (ROS). This surge in ROS levels casts a shadow on the healing landscape, slowing down the intricate dance of tissue renewal [22]. Lavandula stoechas enters this stage as a potential agent, offering a harmonizing presence. Its antioxidative and anti-inflammatory properties seem to wield influence over these cellular regenerations, tilting the balance towards a more favorable healing trajectory. In doing so, Lavandula stoechas addresses the prolonged inflammatory phase, curbing the excessive release of inflammatory cytokines and their potential downstream effects. By quelling the overproduction of ROS, Lavandula stoechas seems to create a conducive environment for healing, one where cellular rejuvenation can flourish. Within this nuanced interplay, the 14th day emerges as a pivotal moment, a juncture where the antioxidative and anti-inflammatory effects of Lavandula stoechas orchestrate a resurgence in healing dynamics. The remarkable outcomes witnessed on the 14th day stand as a testament to the intricate modulation of inflammatory cascades, guided by the gentle touch of Lavandula stoechas' properties. The outcomes on the 14th day lay bare the intricate spectrum of healing processes. The elevation of collagen, hair follicles, collagenization, epithelialization, and fibroblasts are harmonious notes in the symphony of tissue rejuvenation, attesting to the potential of Lavandula Stoechas as a catalyst in managing these rejuvenating effects.

 On the 21st day, a conspicuous upshot of wound healing, surpassing 90%, was discerned in the DM-Lavandula Stoechas, DM-Saline, and non-DM-Lavandula Stoechas groups. This phase marked the culmination of an intricate sequence of reparative processes, manifesting in discernible alterations reflective of the intricate interplay of cellular dynamics. The DM-Lavandula Stoechas group exhibited conspicuous reductions in erythema, mononuclear cell count, dermal edema, and intraepithelial edema, accompanied by a discernible elevation in the abundance of hair follicles. Concurrently, the non-DM-Lavandula Stoechas group demonstrated more diminished levels of erythema and mononuclear cell density. These observations imply Lavandula stoechas' potential to modulate inflammatory markers and foster regenerative markers. The pronounced attenuation in parameters indicative of inflammation within the groups - namely, erythema, mononuclear cell count, dermal edema, and intraepithelial edema - is indicative of a trajectory approaching the resolution of the inflammatory phase. These compelling alterations, reflective of a subdued inflammatory milieu, align with prior research [19] and suggest that Lavandula stoechas' application is instrumental in attenuating inflammatory responses, thereby expediting the progression toward the latter stages of wound healing. The heightened abundance of hair follicles within the DM-Lavandula Stoechas group bears significance as a hallmark of the wound healing process, aligning with previous research [23].

In light of these comprehensive findings, Lavandula stoechas emerges as a promising contender for effective wound healing in the context of diabetes. Nevertheless, the intricate mechanisms underpinning this potential warrant further elucidation. This study paves the way for larger-scope investigations that unravel Lavandula stoechas' journey within the wound bed and its systemic influence.

Limitations

While the current study delved into Lavandula stoechas' impact on wound healing, the exact mechanism remains veiled in complexity. Moreover, the absence of a group that solely isolated the effect of the dressing introduces another element of complexity, potentially leading to alternative interpretations of the results. Additionally, oxidative stress was not assessed in this study, which could have enriched the understanding of Lavandula stoechas' effects.

## Conclusions

In conclusion, our study signifies Lavandula stoechas' potency in bolstering wound healing within the realm of diabetes. This botanical marvel exhibited its prowess in accelerating wound healing, irrespective of diabetic status. These results showed that the H1 hypothesis in this study was confirmed. We propose that larger-scale studies and mechanistic investigations are integral to comprehensively harness the potential of Lavandula Stoechas. Furthermore, isolating the dressing effect from Lavandula stoechas' impact warrants dedicated exploration. This study marks a stepping stone toward a future where Lavandula stoechas' remarkable attributes converge to transform diabetic foot wound management.

The efficacy of Lavandula stoechas against other bacterial strains still needs to be tested in future studies. The efficacy of Lavandula stoechas against other bacterial strains still needs to be tested in future studies. Also, we suggest conducting human clinical experiments to investigate the effect of Lavandula stoechas on wound healing in diabetic individuals.
